# Spatial and temporal expression of surfactant proteins in hyperoxia-induced neonatal rat lung injury

**DOI:** 10.1186/1471-2466-6-8

**Published:** 2006-04-18

**Authors:** Simone AJ ter Horst, Margot Fijlstra, Sujata Sengupta, Frans J Walther, Gerry TM Wagenaar

**Affiliations:** 1 Department of Pediatrics, Division of Neonatology, Leiden University Medical Center, J6-S, Albinusdreef 2, 2333 ZA Leiden, The Netherlands; 2 Los Angeles Biomedical Research Institute at Harbor-UCLA Medical Center, 1124 West Carson Street, Bldg F-5 South, Torrance, California 90502, USA

## Abstract

**Background:**

Bronchopulmonary dysplasia, a complex chronic lung disease in premature children in which oxidative stress and surfactant deficiency play a crucial role, is characterized by arrested alveolar and vascular development of the immature lung. The spatial and temporal patterns of expression of surfactant proteins are not yet fully established in newborn infants and animal models suffering from BPD.

**Methods:**

We studied the mRNA expression of surfactant proteins (SP) A, -B, -C and -D and Clara cell secretory protein (CC10) with RT-PCR and in situ hybridization and protein expression of CC10, SP-A and -D with immunohistochemistry in the lungs of a preterm rat model, in which experimental BPD was induced by prolonged oxidative stress.

**Results:**

Gene expression of all surfactant proteins (SP-A, -B, -C and -D) was high at birth and initially declined during neonatal development, but SP-A, -B, and -D mRNA levels increased during exposure to hyperoxia compared to room-air controls. Peak levels were observed in adult lungs for SP-A, SP-C and CC10. Except for SP-A, the cellular distribution of SP-B, -C, -D and CC10, studied with in situ hybridization and/or immunohistochemistry, did not change in room air nor in hyperoxia. Exposure to normoxia was associated with high levels of SP-A mRNA and protein in alveolar type 2 cells and low levels in bronchial Clara cells, whereas hyperoxia induced high levels of SP-A expression in bronchial Clara cells.

**Conclusion:**

The increased expression of SP-A mRNA under hyperoxia can be attributed, at least in part, to an induction of mRNA and protein expression in bronchial Clara cells. The expanded role of Clara cells in the defence against hyperoxic injury suggests that they support alveolar type 2 cell function and may play an important role in the supply of surfactant proteins to the lower airways.

## Background

Bronchopulmonary dysplasia (BPD) is a chronic lung disease that continues to be a leading cause of mortality and morbidity in preterm infants with respiratory distress syndrome (RDS) despite improved ventilation support and surfactant therapy. BPD is defined clinically by the need for extra oxygen at 36 weeks of gestation, and mainly affects infants born at less than 30 weeks of gestation with a birth weight less than 1,200 g [1,2]. This multifactorial chronic lung disease is characterized by decreased alveolarization and abnormal vascularization and associated with surfactant deficiency, oxidative stress, barotrauma, inflammation, alveolar fibrin deposition, nutrition, and genetic background [1,3]. The (in)balance between initiating factors and host characteristics probably determines whether BPD will occur in a preterm infant. In animal models, including premature baboons, neonatal mice and rats, exposure to hyperoxia results in chronic lung disease which closely resembles BPD in preterm infants [4-9].

Surfactant deficiency and immaturity are pivotal risk factors for developing RDS and, subsequently, BPD in preterm infants [10]. Surfactant therapy dramatically improves the pathophysiology of BPD in preterm infants [11] and attenuates injury, induced by oxidative stress in rodents [12-15]. Various studies have reported the expression of surfactant proteins and CC10 during normal development and under pathological conditions [16-22], but the temporal and spatial distribution patterns of the mRNAs of SP-A, -B, -C and -D and CC10 during normal postnatal lung development and in prolonged hyperoxia in the rat are not fully established. In our laboratory we investigate the pathophysiology of bronchopulmonary dysplasia (BPD) to develop new treatment modalities for this chronic lung disease, using the hyperoxia-exposed preterm rat as a model for experimental BPD [9,23]. In this study we quantified mRNA expression of the surfactant protein genes and CC10 during normal rat lung development and prolonged exposure to hyperoxia with RT-PCR and followed the temporal and spatial distribution with in situ hybridization. We found that all surfactant proteins show a high level of expression at birth and decline during neonatal development in normoxia, whereas the expression of SP-A, -B and -D, but not SP-C, increases in hyperoxia. CC10 expression was not different under both experimental conditions. Peak levels were observed in adult lungs for SP-A, SP-C and CC10. The increased expression of SP-A mRNA during exposure to hyperoxia could be attributed to an induction of SP-A expression in bronchial epithelial Clara cells.

## Methods

### Animals

Timed-pregnant and 6 months old male Wistar rats were kept in a 12 h dark-light cycle and fed a standard chow diet (Special Diet Services, Witham, Essex, United Kingdom) ad libitum. Breeding pairs were allowed access for one hour at the day female Wistar rats showed very specific sexual behaviour: lordosis, hopping and air-flapping. After a gestation of approximately 21^1/2 ^days pregnant Wistar rats were killed by decapitation (spontaneous birth occurs 22 days after conception) and pups delivered by hysterectomy through a median abdominal incision to ensure that the delay in birth between the first and the last pup is only 5 minutes. Immediately after birth, pups were dried and stimulated. Pups of 2–3 litters, with a maximum of 12 pups, were pooled and exposed to 100% oxygen in a transparent 50×50×70 cm Plexiglas chamber for 10 days. In this way influences of the birth process within and between litters can be avoided and exposure to hyperoxia can be started within 30 minutes after birth. Pups were fed by lactating fosters. To avoid oxygen toxicity of the foster mothers, two groups of 5 pups exposed to room air were included and used to generate data on room air-exposed controls. Foster mothers were rotated daily between the 3 cages. The research protocol was approved by the Institutional Animal Care and Use Committee of the Leiden University Medical Center.

### Tissue preparation

Pups were anesthesized with an intraperitoneal injection of ketamine (50 mg/kg bodyweight, Ketanest-S™ Parke-Davis/Pfizer, New York, NY, USA) and xylazine (50 mg/kg bodyweight, Rompun™, Bayer AG, Leverkusen, Germany). To avoid post-mortem fibrin deposition in the lungs, heparin (100 units, Leo Pharma, Breda, the Netherlands) was injected intraperitoneally. After 5 min, pups were exsanguinated by transection of the abdominal blood vessels. The thoracic cavity was opened and the lungs were removed, snap-frozen in liquid nitrogen and stored at -80°C until use for real-time RT-PCR. For histology studies and in situ hybridization, the trachea was cannulated (Bioflow 0.6 mm i.v. catheter, Vygon, Veenendaal, the Netherlands) and the lungs were fixed in situ via the trachea cannula with buffered formaldehyde (4% paraformaldehyde in phosphate buffered saline, pH 7.4) at 25 cm H_2_O pressure for 3 min. Lungs were removed, fixed additionally in formaldehyde for 24 h at 4°C, and embedded in paraffin after dehydration in a graded alcohol series and xylene.

### Real-time RT-PCR

Total RNA was isolated from lung tissue homogenates using guanidium-phenol extraction (RNAzol™, Campro Scientific, Veenendaal, the Netherlands). Briefly, after tissue homogenization in RNAzol B, RNA was isolated using phenol-chloroform extraction and isopropanol precipitation. The RNA sample was dissolved in RNase-free water and quantified spectrophotometrically. The integrity of the RNA was studied by gel electrophoresis on a 1% agarose gel, containing ethidium bromide. Samples showing degradation of ribosomal RNA by visual inspection under UV-light were discarded. First strand cDNA synthesis was performed with the SuperScript™ Choice System (Life Technologies, Breda, the Netherlands) by mixing 2 μg total RNA with 1 μg Oligo(dT)12–18 primer in a total volume of 10 μl. After heating the mixture at 70°C for 10 min, a solution containing 50 mM Tris-HCl (pH 8.3), 75 mM KCl, 3 mM MgCl_2_, 10 mM DTT, 0.5 mM dNTPs, 0.5 μl RNase inhibitor, and 200 U Superscript Reverse Transcriptase was added, resulting in a total volume of 20 μl. This mixture was incubated at 42°C for 1 h, total volume was adjusted to 100 μl with RNase-free water, and stored at -80°C until further use. For real-time quantitative PCR 1 μl first strand cDNA, diluted 1:10 in RNase-free water, was used in a total volume of 25 μl, containing 12.5 μl 2 × SYBR Green PCR Master Mix (Applied Biosystems, Foster City, CA, USA) and 200 ng of each primer. Primers, designed with the Primer Express™ software package (Applied Biosystems, Foster City CA, USA), are listed in Table 1. PCR reactions, consisting of 95°C for 10 min (1 cycle), 94°C for 15 sec and 60°C for 1 min (40 cycles), were performed on an ABI Prism 7700 Sequence Detection System (Applied Biosystems, Foster City CA, USA). Data were analyzed with the ABI Prism 7700 Sequence Detection System version 1.9 software and quantified using the comparative threshold cycle method with β-actin as a housekeeping gene reference [24]. We studied at least 6 animals per experimental group.

**Table 1:** Sequences of oligonucleotides used as forward and reverse primers for real-time RT-PCR

Gene product	Forward primer	Reverse primer
SP-A	5'-TACCAGAGCAGGAGGCAACA-3'	5'-CAATACTTGCAATGGCCTCGTT-3'
SP-B	5'-CCATCCCTCTGCCCTTCTG-3'	5'-CACCCTTGGGAATCACAGCTT-3'
SP-C	5'-TCCCAGGAGCCAGTTTCG-3'	5'-CACGATGAGAAGGCGTTTGA-3'
SP-D	5'-AAATCTTCAGGGCGGCAAA-3'	5'-GGCCTGCCTGCACATCTC-3'
CC10	5'-TTACAACATCAGCCCACATCTACA-3'	5'-TGTGATGCCGATCTTCATGGT-3'
β-actin	5'-TTCAACACCCCAGCCATGT-3'	5'-AGTGGTACGACCAGAGGCATACA-3'

### In situ hybridization

Paraffin sections (7 μm) from the left upper lobe were cut and mounted onto SuperFrost plus coated slides (Menzel-Gläzer, Braunschweig, Germany). The in situ hybridization procedure was based on the method described by Wilkinson [25]. Sections were deparaffined, digested with proteinase K (Roche, Almere, The Netherlands), postfixed in 4% paraformaldehyde, treated with 0.2 M HCl to block endogenous alkaline phosphatase activity, acetylated with acetic anhydride dissolved in triethanolamine and hybridized with digoxygenine-labeled probe in hybridization buffer overnight at 55 °C. cRNA probe concentrations were 20 ng/section for CC10, SP-B and SP-C, 30 ng/section for SP-A, and 40 ng/section for SP-D. The hybridization buffer contained 9/10 (volume) hybridization mixture: 50% formamide, 2 × SSC, 1 × Denhardt's solution and 10% dextran sulphate and 1/10 (volume) probe solution: 0.1–1 ng/μl cRNA probe and 1 μg/μl yeast RNA in distilled water. After hybridization, high stringency washing was performed in 50% formamide/2 × SSC at 55 °C. Sections were treated with RNase A, rinsed in 2 × SSC, blocked with lamb serum (Invitrogen, Paisley, England) and incubated with anti-digoxygenine-alkaline phosphatase conjugated Fab fragments (Roche, Almere, The Netherlands) for 2 hours. Sections were washed extensively with Tris-buffered saline (100 mM Tris, 150 mM NaCl, pH 7.5) and incubated with NBT/BCIP solution (Roche, Almere, The Netherlands), dissolved in 6% polyvinyl alcohol, overnight in the dark. The staining reaction was terminated with TE-buffer (10 mM Tris/HCl, 1 mM EDTA, pH 8.0). Subsequently, sections were counterstained with nuclear fast red, dehydrated with an ethanol series, and mounted in Euparal. As a negative control RNase pretreated sections were used prior to hybridization, which did not give any staining at all. We studied a minimum of four animals per time-point.

Digoxygenine-labeled anti-sense cRNA probes to rat SP-A, SP-B, SP-C, SP-D and CC10 were made by in vitro transcription using pBluescript (SP-A), pGEM 4Z (SP-D) and pCR4-TOPO (SP-B, SP-C and CC10) as vectors, containing a 662 bp rat SP-A cDNA fragment [26], a 998 bp SP-B fragment, a 521 bp SP-C fragment, a full length SP-D fragment [27] and a 416 bp CC10 fragment. Primers used to generate PCR fragments for SP-B, SP-C and CC10 from a first strand cDNA rat lung (10 days after birth) sample (see real-time RT-PCR section) were designed with primer3 software and are listed in Table 2. PCR reactions, consisting of 95°C for 5 min (1 cycle), 95°C for 1 min, 60°C for 1 min and 72 °C for 1 min (40 cycles) and 72°C for 30 min were performed in the presence of Amplitaq gold (Invitrogen, Paisley, England) on a Perkin Elmer GeneAmp PCR system 9600 (Perkin Elmer, Boston, MA, USA). PCR fragments were subsequently cloned into pCR4-TOPO vector exactly as described by the manufacturer (Invitrogen, Breda, The Netherlands) and checked by restriction fragment analysis and sequencing.

**Table 2:** Sequences of oligonucleotides used as forward and reverse primers for PCR

Gene product	Forward primer	Reverse primer
SP-B	5'-GCCATGGCCAAGTTACATCT-3'	5'-TGCTCACACTTTTGCCTGTC-3'
SP-C	5'-TACTCGACAGGTCCCAGGAG-3'	5'-AGCTCTCCACACAAGGTGCT-3'
CC10	5'-CAACATCAGCCCACATCTACA-3'	5'-TTGGTGTAGGGAGGTCAAGG-3'

### Immunohistochemistry

Paraffin sections (5 μm) from the left upper lobe were cut and mounted onto SuperFrost plus coated slides (Menzel-Gläzer, Braunschweig, Germany). After deparaffinization sections were incubated with 0.3% H_2_O_2 _in methanol to block endogenous peroxidase activity. After a graded alcohol series, sections were pretreated for antigen retrieval prior to antibody incubation. For the detection of CC10 and SP-A sections were boiled in 0.01 M sodium citrate (pH 6.0) for 10 min. For the detection of SP-D sections were digested with proteinase K, 20 mg/ml in a buffer containing 100 mM Tris/HCl, 50 mM EDTA, pH 8.0 for 30 minutes. Hereafter sections were incubated overnight with rabbit anti-rat CC10 (C5828-03, United States Biological, Swampscott, MA), rabbit anti-rat SP-A and mouse anti-rat SP-D (HM3022, Hycult Biotechnology, Uden, The Netherlands) and stained with EnVision-HRP (Dako, Glostrup, Denmark), using NovaRed (Vector, Burlingame, CA, USA) as chromogenic substrate. Sections were counterstained briefly with hematoxylin. SP-B and SP-C protein expression could not be evaluated due to a limited supply of anti-rat SP-B and high background activity in incubations with anti-rat SP-D.

### Statistical analysis

Values are expressed as mean ± SEM. Differences between groups were analyzed with the Student's *t*-test. P-values < 0.05 were considered statistically significant.

## Results

### RT-PCR

**Figure 1:**
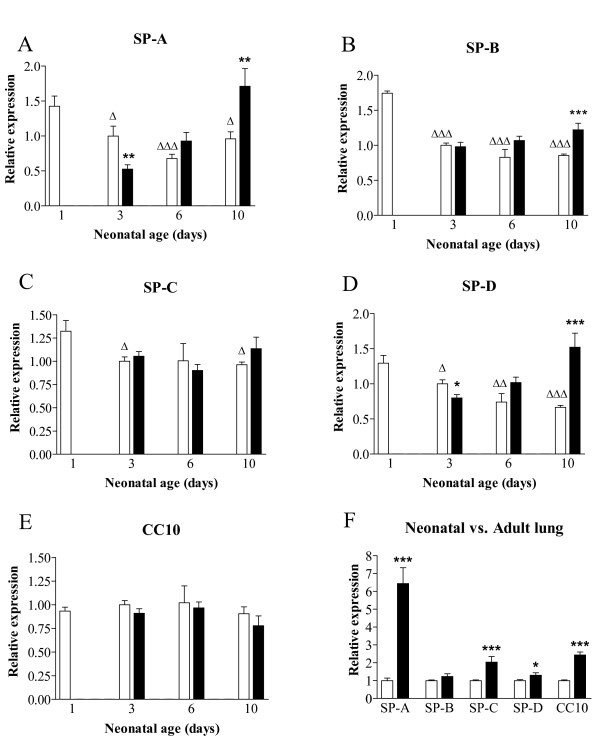
Relative mRNA expression, determined with RT-PCR, of surfactant proteins A (A), -B (B), -C (C) and -D (D) and Clara cell secretory protein CC10 (E) in oxygen-exposed (black bars) and control pups (white bars) in neonatal lungs on days 1, 3, 6, and 10. In panel F adult mRNA expression is depicted by black bars and neonatal expression in room air-exposed controls on day 3 by white bars. Data are expressed as mean ± SEM of 9 (day 1) or 6 (days 3, 6 and 10, and 6 months) rats. *: p < 0.05; **: p < 0.01, ***: p < 0.001 versus age-matched controls (panels A-E) or versus day 3 room air (panel F). Δ: p < 0.05, ΔΔ: p < 0.01, ΔΔΔ: p < 0.001 versus day 1.

mRNA expression of SP-A, SP-B, SP-C, SP-D and CC10 was studied in lungs of hyperoxia-exposed pups on neonatal days 3, 6 and 10, and age-matched controls on days 1, 3, 6 and 10, and at 6 month of age with normal pulmonary development (Figure 1). To exclude the effects of birth on gene expression, mRNA expression on neonatal day 3 in room air exposed controls was used as a reference value. The collectins SP-A (panel A) and SP-D (panel D) showed similar expression patterns during normal neonatal lung development and in hyperoxia. Highest expression in control lungs was observed at birth and decreased gradually thereafter. SP-A decreased 2.1-fold (p = 0.0001) and 1.5-fold (p = 0.0169) on days 6 and 10, respectively, and SP-D decreased 1.9-fold (p < 0.0001) on day 10, compared to expression at birth. During exposure to hyperoxia, SP-A and SP-D expression on day 3 decreased 1.9-fold (p = 0.0038) and 1.25-fold (p = 0.0174), respectively, compared to age-matched controls. Thereafter, expression of SP-A and SP-D gradually increased in hyperoxia, resulting in an 1.8-fold (p = 0.0066) and a 2.3-fold (p = 0.0001) increase on day 10 for SP-A and SP-D, respectively, compared to age-matched controls, indicating that the magnitude of the effect of hyperoxia on gene expression is related with the duration of oxygen exposure.

The hydrophobic surfactant proteins SP-B (panel B) and SP-C (panel C) showed a similar expression pattern during normal neonatal development. Expression was highest at birth and decreased thereafter with a 2.0-fold (p < 0.0001) decrease for SP-B and an 1.4-fold decrease (p = 0.0104) for SP-C on day 10. In hyperoxia, SP-B showed an 1.4-fold (p = 0.0009) increase in expression on day 10, compared to age-matched controls. Expression of SP-C did not change in hyperoxia. Expression of CC10 mRNA did not change during normal neonatal lung development and in hyperoxia (panel E).

In adult lung mRNA expression of SP-A, SP-C, SP-D and CC10 was increased 6.4-fold (p < 0.0001), 2.0-fold (p = 0.0007), 1.3-fold (p = 0.0446) and 2.4-fold (p < 0.0001), respectively, compared with mRNA expression on day 3 in room air (panel F).

### In situ hybridization and immunohistochemistry

**Figure 2:**
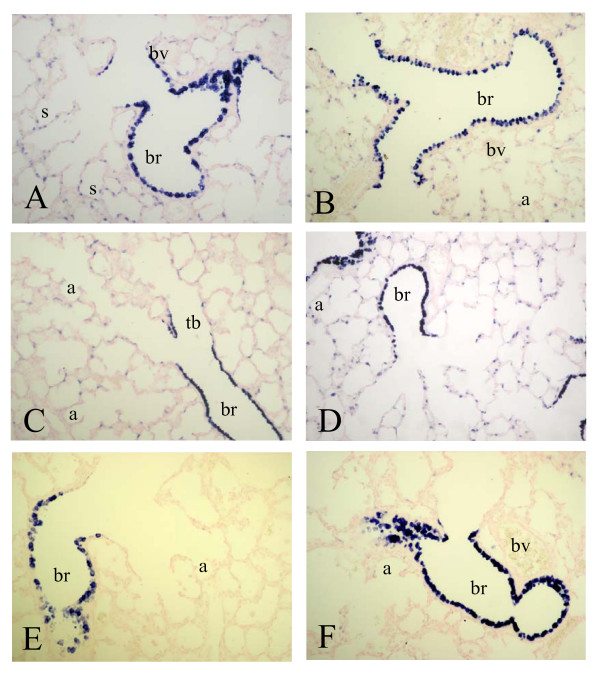
mRNA expression of Clara cell secretory protein, determined with in situ hybridization on formaldehyde fixed paraffin sections of rat lungs during normal postnatal development at birth (A), day 6 (C), day 10 (E) and 6 months (B) and after exposure to hyperoxia on day 6 (D) and 10 (F) at a 200-fold magnification. a = alveolus, br = bronchus, bv = blood vessel, s = saccule, tb = terminal bronchus.

To investigate whether differential mRNA expression of SP-A, -B, -C, -D and CC10 could be attributed to changes in gene expression at the cellular level we studied the spatial and temporal expression of these genes during normal neonatal lung development until adulthood and in experimental BPD at the mRNA level with in situ hybridization (SP-A, -B, -C, -D and CC10) and at the protein level with immunohistochemistry (SP-A, -D and CC10). Expression was studied in the lung during the first two weeks after birth, in which the transition from the saccular to the alveolar developmental stage takes place, in hyperoxia-exposed pups on neonatal days 1, 3, 6 and 10, in age-matched room-air exposed controls, including day 14, and in adult rat lungs. As a positive control for Clara cells we studied the expression of CC10 mRNA (Figure 2) and protein (Figure 7) and used this as a reference for surfactant protein expression in bronchial epithelial Clara cells. From birth (Figure 2, panel A) onward CC10 mRNA was predominantly, but not exclusively, expressed at high levels in the bronchial epithelium in non-ciliated cuboidal cells, consistent with the morphological definition of Clara cells [28], but also at a low level in alveolar type 2 cells. This pattern of mRNA expression did not change during postnatal development on days 3, 6 (panel C) and 10 (panel E) until adulthood (panel B) and after exposure to hyperoxia on days 3, 6 (panel D) and 10 (panel F). In contrast to its mRNA CC10 protein (Figure 7) was absent in alveolar type 2 cells and was exclusively expressed in bronchial epithelial Clara cells from birth onward (panel A). This pattern of protein expression did not change during postnatal development on days 3, 6 (panel C) and 10 (panel E) until adulthood (panel B) and after exposure to hyperoxia on days 3, 6 (panel D) and 10 (panel F).

**Figure 3:**
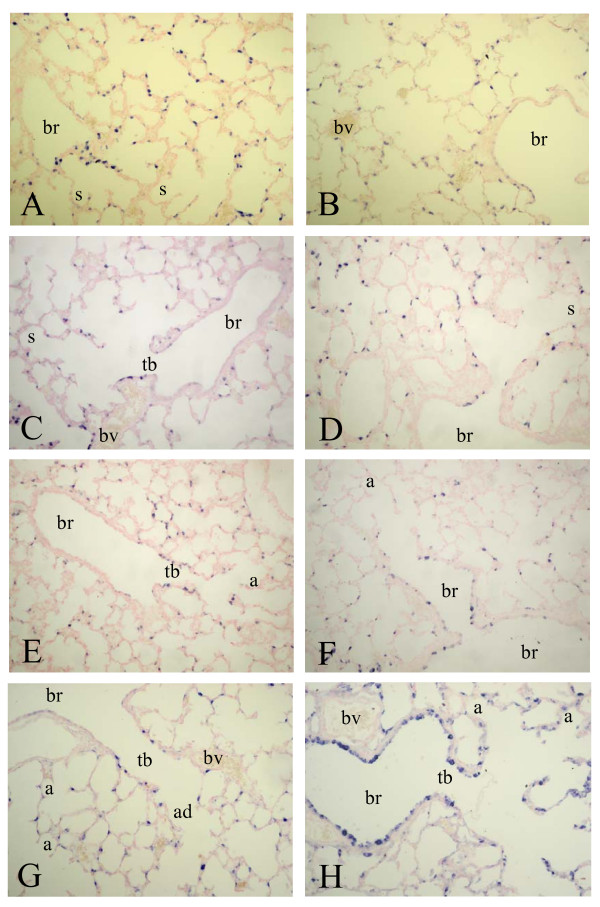
mRNA expression of surfactant protein A, determined with in situ hybridization on formaldehyde fixed paraffin sections of rat lungs during normal postnatal development at birth (A), day 3 (C), day 6 (E) day 10 (G) and 6 months (B) and after exposure to hyperoxia on day 3 (D), 6 (F) and 10 (H) at a 200-fold magnification. a = alveolus, ad = alveolar duct, br = bronchus, bv = blood vessel, s = saccule, tb = terminal bronchus.

SP-A mRNA was expressed from birth (Figure 3, panel A) until day 10 (panel G) in alveolar type 2 cells and in the epithelial lining of terminal bronchi and the alveolar duct, but was absent in the epithelium of more upstream localized bronchi. A relatively low level of SP-A expression was observed in bronchial epithelial cells at two weeks after birth and in adult lungs (panel B). Hyperoxia resulted in an induction of SP-A expression in the bronchial epithelium in Clara cells from day 6 onward (panel F), resulting in a high level of expression in alveolar type 2 cells and, in contrast to age-matched controls, in the epithelial lining of the bronchi (Clara cells) on day 10 (panel H). SP-A protein expression at birth was low. Similar to its mRNA SP-A protein (Figure 8) was expressed from day 3 (panel A) until day 10 (panel E) in alveolar type 2 cells and in epithelial cells of the alveolar duct and terminal bronchi. Hereafter SP-A protein was expressed in alveolar type 2 cells and in bronchial epithelial Clara cells (not shown). At day 3 SP-A expression was similar in hyperoxia-exposed lungs (panel B) and in age-matched controls (panel A). From day 6 (panel D) onward hyperoxia also resulted in an induction of SP-A expression in the bronchial epithelium in Clara cells, resulting in a high level of expression in alveolar type 2 cells and, in contrast to age-matched controls, in the epithelial lining of the bronchi (Clara cells) on day 10 (panels D and F).

**Figure 4:**
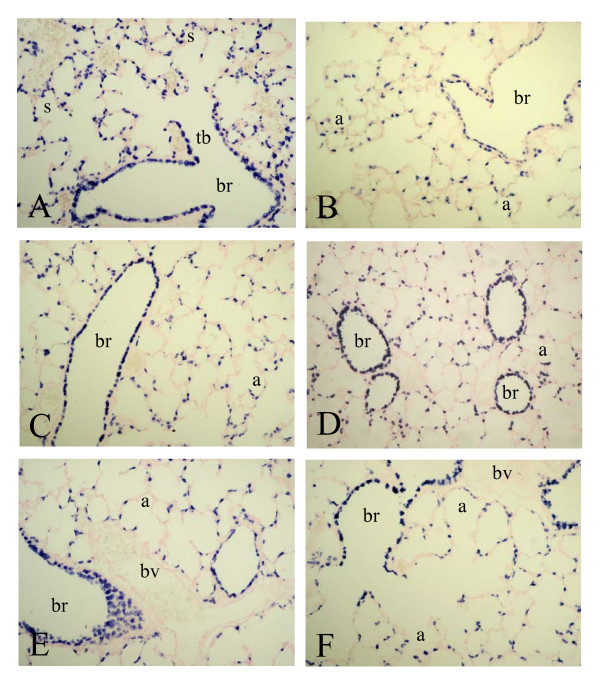
mRNA expression of surfactant protein B, determined with in situ hybridization on formaldehyde fixed paraffin sections of rat lungs during normal postnatal development at birth (A), day 6 (C), day 10 (E) and 6 months (B) and after exposure to hyperoxia on day 6 (D) and 10 (F) at a 200-fold magnification. a = alveolus, br = bronchus, bv = blood vessel, s = saccule, tb = terminal bronchus.

At birth a high level of expression of SP-B mRNA was observed in alveolar type 2 cells and in the bronchial epithelium in Clara cells (Figure 4, panel A). Hereafter, SP-B expression did not change significantly during normal neonatal development on days 6 (panel C) and 10 (panel E) until adulthood (panel B) and after exposure to 100% oxygen on days 6 (panel D) and 10 (panel F).

**Figure 5:**
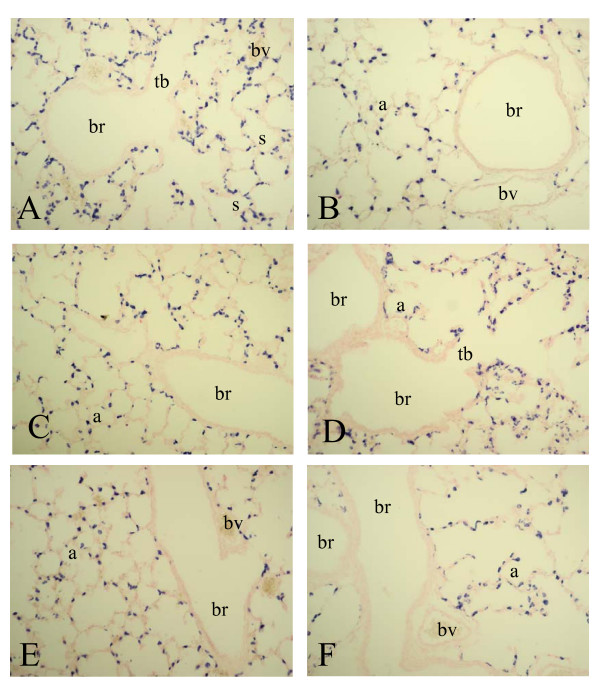
mRNA expression of surfactant protein C, determined with in situ hybridization on formaldehyde fixed paraffin sections of rat lungs during normal postnatal development at birth (A), day 6 (C), 10 (E) and 6 months (B) and after exposure to hyperoxia on day 6 (D) and 10 (F) at a 200-fold magnification. a = alveolus, br = bronchus, bv = blood vessel, s = saccule, tb = terminal bronchus.

From birth onward SP-C mRNA (Figure 5, panel A) was exclusively present in the alveolar epithelium, including the alveolar duct and alveolar type 2 cells, but was absent in the bronchial epithelium on days 6 (panel C) and 10 (panel E) until adulthood (panel B). The SP-C expression pattern did not change during exposure to hyperoxia for 6 days (panel D) and 10 days (panel F).

**Figure 6:**
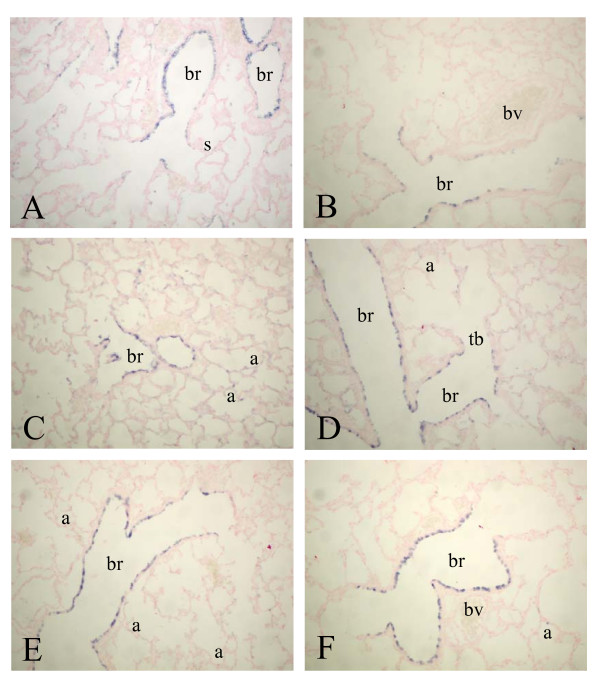
mRNA expression of surfactant protein D, determined with in situ hybridization on formaldehyde fixed paraffin sections of rat lungs during normal postnatal development at birth (A), day 6 (C), 10 (E) and 14 (B) and after exposure to hyperoxia on day 6 (D) and 10 (F) at a 200-fold magnification. a = alveolus, br = bronchus, bv = blood vessel, s = saccule, tb = terminal bronchus.

Expression of SP-D mRNA was almost complementary to that of the other collectin SP-A and CC10, showing a high level of expression in bronchial Clara cells and a very low level of expression in alveolar type 2 cells after birth (Figure 6, panel A) until day 14 (panel B). This pattern of mRNA expression in the lung did not change after exposure to 100% oxygen (day 10, panel F). In adult lungs SP-D mRNA expression was below the detection level (not shown). At birth SP-D protein expression was relatively low and was observed in alveolar type 2 cells and in bronchial Clara cells, showing highest expression in alveolar type 2 cells (Figure 9, panel A). Hereafter, SP-D protein expression was similar as its mRNA expression, showing a high level of expression in bronchial epithelial Clara cells and to a lesser extent in alveolar type 2 cells. This pattern did not change during normal post-natal development until adulthood and after hyperoxia. However, hyperoxia reduced the overall expression of SP-D, resulting in a very low expression on day 10 in both alveolar type 2 cells and in bronchial epithelial cells (panel F).

**Figure 7:**
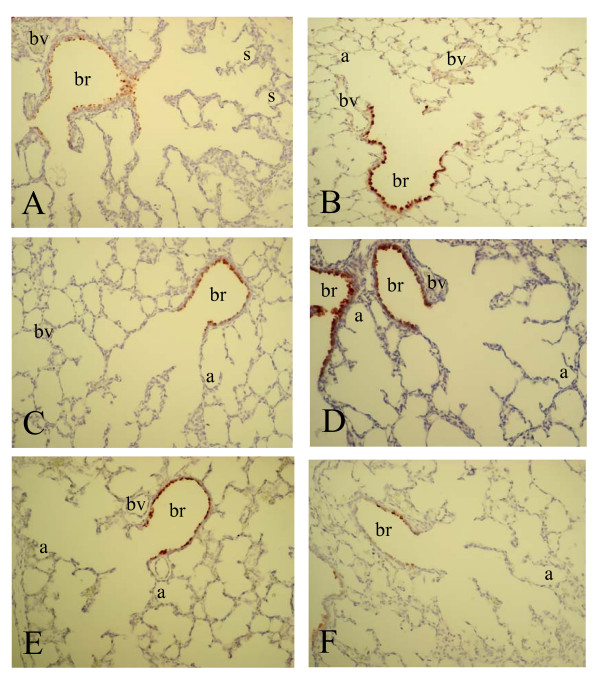
Immunohistochemical staining of Clara cell secretory protein on formaldehyde fixed paraffin sections of rat lungs during normal postnatal development at birth (A), day 6 (C), day 10 (E) and 6 months (B) and after exposure to hyperoxia on day 6 (D) and 10 (F) at a 200-fold magnification. a = alveolus, br = bronchus, bv = blood vessel, s = saccule, tb = terminal bronchus.

**Figure 8:**
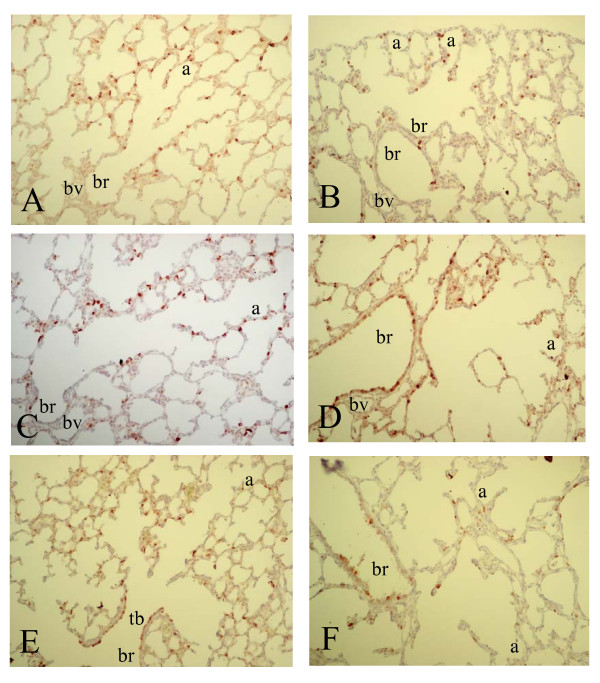
Immunohistochemical staining of surfactant protein A on formaldehyde fixed paraffin sections of rat lungs during normal postnatal development on day 3 (A), day 6 (C) and day 10 (E) and after exposure to hyperoxia on day 3 (B), 6 (D) and 10 (F) at a 200-fold magnification. a = alveolus, br = bronchus, bv = blood vessel, s = saccule, tb = terminal bronchus.

**Figure 9:**
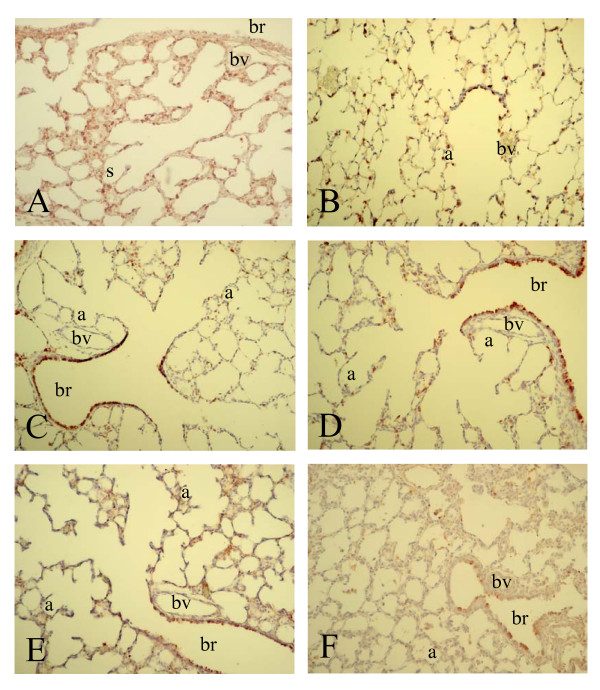
Immunohistochemical staining of surfactant protein D on formaldehyde fixed paraffin sections of rat lungs during normal postnatal development at birth (A), day 6 (C), day 10 (E) and 6 months (B) and after exposure to hyperoxia on day 6 (D) and 10 (F) at a 200-fold magnification. a = alveolus, br = bronchus, bv = blood vessel, s = saccule, tb = terminal bronchus.

## Discussion

Using a preterm rat model we quantified mRNA expression and investigated the temporal and spatial expression of surfactant proteins (SP) A, B, C and D, and Clara cell secretory protein (CC10) at the mRNA level and SP-A, -B and CC10 at the protein level during normal neonatal pulmonary development and in experimental BPD induced by oxidative stress. mRNA levels of all surfactant proteins were high at birth and increased after prolonged exposure to hyperoxia, compared to age-matched controls. Peak levels were observed in adult lungs for SP-A and SP-C. Expression of CC10 mRNA did not change during normal neonatal lung development and exposure to hyperoxia, but peaked in adult lung. An upregulation of surfactant protein expression at the mRNA level after prolonged hyperoxia in premature rat pups confirms the results obtained for SP-A, -B, -C and/or -D in neonatal rabbits [18,20], neonatal rats [21], adult rabbits [19,29] and adult rats [16,17]. Our data confirm the observation by White and co-workers [21] that hyperoxia increases the tissue expression of surfactant protein mRNAs in newborn rats. However, earlier (at premature birth, at least 12 hours ahead of the normal birthing period by caesarean section, versus 48 hours of age) and longer (up to 10 days instead of 8 days) exposure to hyperoxia in this study provides new details on the spatial location of the increase in cellular expression of SP-A and SP-D. During hyperoxia, SP-A expression mainly increases in Clara cells, whereas the location of SP-D expression does not change. The initial decrease in mRNA expression of the collectins after 3 days of exposure to hyperoxia, compared to room-air exposed controls, supports the findings in adult rats, in which a temporary decrease in expression of SP-A, -B and -C was observed after 12 hours of exposure to 95% oxygen [30]. The cellular expression patterns of SP-A and SP-D mRNA during neonatal development and after exposure to hyperoxia were confirmed at the protein level, suggesting regulation of gene expression at a transcriptional level. High mRNA expression of CC10 in Clara cells was also confirmed at the protein level, but the relatively low staining of its mRNA in alveolar type 2 cells could not be established at the protein level, suggesting that our immunohistochemical staining procedure of CC10 is below the detection level in these cells.

The remarkable upregulation of SP-A and SP-D in preterm rats exposed to hyperoxia matches the findings in ventilated preterm baboons [31] and lambs [32]. In baboons of 140 days of gestation and ventilated with 100% oxygen for 10 days, the tissue concentration of SP-A was five and of SP-D sixteen times greater than in normal adults, but this was not associated with an increased total collectin pool in the lavage, suggesting regulation at the translational level and inhibition of secretion [31]. In preterm lambs, postnatal changes in surfactant protein gene expression as a result of hyperoxia are dependent on gestational age [32]. Exposure to hyperoxia had no effect on SP-A, B, and C mRNA levels in lambs at 120 days of gestation, but increased SP-A and B mRNA, but not SP-C mRNA, levels in more mature preterm lambs [32], not unlike the findings in this study.

Although, the collectins SP-A and -D show a similar gene expression profile in whole lungs during normal neonatal development and exposure to hyperoxia, the cellular expression pattern of both collectins is almost complementary during the first 10 days after birth: SP-A is present at high levels in alveolar type 2 cells and in epithelial cells of terminal bronchi, whereas SP-D is expressed at high levels in bronchial epithelial Clara cells and at low levels in alveolar type 2 cells. The increased expression of SP-A during prolonged hyperoxia was, at least in part, ascribed to an induction of SP-A mRNA and protein in the bronchial epithelium, whereas the expression pattern of SP-D did not change significantly after prolonged exposure to 100% oxygen. A similar induction of SP-A mRNA expression in bronchial epithelial cells after exposure to hyperoxia has been reported in adult rabbits [19,29] and in terminal bronchi in newborn rabbits [20], whereas increased SP-A expression after hyperoxia was attributed to increased expression in alveolar type 2 cells in newborn [21] and adult rats [17]. These data strongly indicate that both alveolar type 2 cells and bronchial Clara cells are involved in increased synthesis and secretion of SP-A after oxygen-induced lung injury.

Data on surfactant protein gene expression in preterm infants ventilated for RDS are not available, although various studies have measured surfactant protein content in bronchoalveolar lavage material. Beresford and Shaw [33] measured surfactant protein concentrations in bronchoalveolar lavage by ELISA in a cohort of 50 preterm infants. SP-A, B, and D concentrations rose significantly during the first postnatal week and lower broncholaveolar lavage SP-B and SP-D, but not SP-A, concentrations were associated with worse clinical prognosis. In a prospective study Merrill et al. [34] analyzed tracheal aspirate samples from 68 infants of 23–30 weeks of gestation who remained intubated for 7–84 days. Most preterm infants requiring continued ventilatory support after 7 days of age experienced a deficiency of SP-B and SP-C. Hallman et al. [35] reported that infants weighing <1,000 g had a strikingly lower SP-A/saturated phosphatidylcholine ratio during the first week of life if they were going to die or develop bronchopulmonary dysplasia. These studies suggest that development of bronchopulmonary dysplasia in preterm infants is associated with reduced concentrations of surfactant proteins in bronchoalveolar lavages. This may not be unlike the situation in ventilated preterm baboons, in whom upregulation of surfactant protein gene expression does not increase the surfactant protein pool in the airways due to regulation at the translational level and/or inhibition of secretion [31]. We did not measure surfactant proteins in lung lavages of the hyperoxia exposed preterm rats, but would expect their concentrations to fall in line with the above described reports.

Early hyperoxia exposure in preterm rats rapidly increases the expression of surfactant proteins in the lung, whereby SP-A expression is expanded in Clara cells while SP-D expression does not shift from its initial production sites. The expanded role of Clara cells in the defence against hyperoxic injury suggests that they support alveolar type 2 cell function and may play an important role in the supply of surfactant proteins to the lower airways.

## Conclusion

• mRNA levels of all surfactant proteins were high at birth and increased after prolonged exposure to hyperoxia, compared to age-matched controls.

• SP-A and SP-C mRNA levels peaked in adult lungs.

• Expression of CC10 mRNA did not change during normal neonatal lung development and exposure to hyperoxia, but peaked in adult lung.

• Similar changes in the cellular expression patterns of CC10, SP-A and SP-D at the mRNA and protein level during normal neonatal development and after exposure to hyperoxia suggests regulation of gene expression at a transcriptional level.

• The cellular expression pattern of both collectins SP-A and SP-D is almost complementary during the first 10 days after birth, despite a similar gene expression profile in whole lungs.

• The increased expression of SP-A under hyperoxia could be attributed, at least in part, to an induction of expression in bronchial Clara cells.

• The expanded role of Clara cells in the defense against hyperoxic injury suggests that they support alveolar type 2 cell function and may play an important role in the supply of surfactant proteins to the lower airways.

## Competing interests

SAJtH, MF, SS, FJW, and GTMW declare that they have no competing interests.

## Authors' contributions

SAJtH and MF carried out the real-time RT-PCR and in situ hybridization studies and drafted the manuscript. SS participated in the in situ hybridization studies. FJW and GTMW conceived of the study, participated in its design and coordination, and helped to write the manuscript. All authors participated in the animal care and tissue preparation and read and approved the final manuscript.

## Pre-publication history

The pre-publication history for this paper can be accessed here:


